# The pathology, phylogeny, and epidemiology of *Echinococcus ortleppi* (G5 genotype): a new case report of echinococcosis in China

**DOI:** 10.1186/s40249-021-00907-3

**Published:** 2021-11-06

**Authors:** Xu Wang, Aiya Zhu, Hongying Cai, Baixue Liu, Gang Xie, Rui Jiang, Ji Zhang, Nanzi Xie, Yayi Guan, Robert Bergquist, Zhenghuan Wang, Yang Li, Weiping Wu

**Affiliations:** 1 National Institute of Parasitic Diseases, Chinese Center for Diseases Control and Prevention (Chinese Center for Tropical Diseases Research); NHC Key Laboratory of Parasite and Vector Biology; WHO Collaborating Centre for Tropical Diseases; National Center for International Research on Tropical Diseases, Shanghai, China; 2 Guizhou Center for Disease Control and Prevention, Guiyang, Guizhou China; 3 People’s Hospital of Anshun City, Anshun, Guizhou China; 4 Anshun Center for Disease Control and Prevention, Anshun, Guizhou China; 5 Geospatial Health Journal, Ingerod, Brastad, Sweden; 6 School of Life Sciences, East China Normal University, Shanghai, China

**Keywords:** Cystic echinococcosis (CE), *Echinococcus ortleppi*, Human, Non-endemic area, China

## Abstract

**Background:**

Cystic echinococcosis (CE), caused by the larval stage of the complex *Echinococcus granulosus* sensu lato (s.l.), is a zoonotic parasitic disease with a high social burden in China. *E. ortleppi* is a species (formerly genotype 5 of *E. granulosus* s.l.) with unique epidemic areas (tropical areas), transmission patterns (mainly cattle origin), and pathological characteristics (large and small hook lengths) compared to other species that cause CE. A 19-year-old female patient in an area with no history of echinococcosis in Guizhou Province, China, was diagnosed with *E. ortleppi* infection in 2019. This study is to understand the source of this human *E.* *ortleppi* infection.

**Methods:**

We performed computer tomography (CT) scans, surgical operation, morphological sectioning, molecular diagnosis, phylogenetic analyses, and epidemiological investigation in Anshun City, Guizhou Province, China in 2019.

**Results:**

The patient presented with intermittent distension and pain in the upper abdomen without other abnormal symptoms. Routine blood examination results were normal. However, abdominal CT revealed a fertile cyst with a diameter of approximately 8 cm, uniform density, and a clear boundary, but without an evident cyst wall in the right lobe of the liver. The cyst was fertile, and phylogenetic analyses revealed that the isolates represented a new *E. ortleppi* genus haplotype. A result of 10‒14 years incubation period with indigenous infection was considered available for the case through the epidemiological survey.

**Conclusions:**

CE due to *E. ortleppi* infection can be confused with other diseases causing liver cysts, resulting in misdiagnosis. A transmission chain of *E. ortleppi* may exist or existed in the past in the previously considered non-endemic areas of echinococcosis in southwestern China.

**Graphic abstract:**

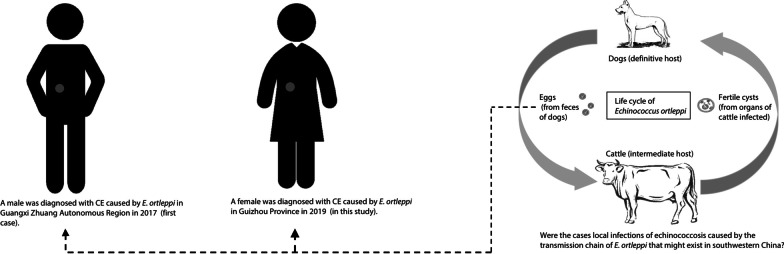

**Supplementary Information:**

The online version contains supplementary material available at 10.1186/s40249-021-00907-3.

## Background

Echinococcosis or hydatidosis is a global, often fatal zoonotic helminthic disease caused by *Echinococcus* sp. (Cestoda: Taeniidae) tapeworm larvae [1]. Intermediate hosts (such as wild herbivores, livestock, and small mammals), as well as accidentally infected humans receive their infections through ingestion of contaminated food and water contaminated by *Echinococcus* eggs from the feces of the definitive hosts (such as dogs and foxes, etc.) [2]. The eggs hatch to release oncospheres in the intestinal tract, which migrate via the blood circulation to other organs of the body, mainly the liver and lungs, where they develop into hydatid cysts [1, 2]. Presently, nine species of *Echinococcus* have been reported globally, with six confirmed zoonoses caused by *E. granulosus*, *E. multilocularis*, *E. vogeli*, *E. oligarthra*, *E. canadensis* and *E. ortleppi*; while there is to date no zoonotic evidence for *E. equinus*, *E. felidis* and *E. shiquicus*, which have only been reported to complete their life cycles in different mammals [3, 4].

The main species infecting humans is *Echinococcus granulosus,* which is globally distributed and generally circulates between dogs and livestock causing cystic echinococcosis (CE) [5]. Echinococcosis remains a significant health problem with a considerable socioeconomic burden in endemic areas, especially in China [5, 6]. Since 1990, several studies focusing on host species, molecular biology, and morphology have posited that *E. granulosus* is a genotype complex—*E. granulosus* sensu lato (s.l.), comprising genotypes G1‒G10, and the so-called lion strain. To date, *E. granulosus* s.l. has been split into five separate species, *E*. *granulosus* sensu stricto (s.s.) (G1/G2/G3 genotypes), *E*. *equinus* (G4 genotype), *E*. *ortleppi* (G5 genotype), *E*. *canadensis* group (G6/G7/G8/G10 genotypes), and *E*. *felidis* (lion strain) [7–11]. In China, G1 is the predominant genotype, with G3 of *E*. *granulosus* s.s. and G6, G7, G8, and G10 of the *E*. *canadensis* group recently reported sporadically [12–16].

*Echinococcus ortleppi* infection was first reported in a herd of cattle in Holland; hence, it was named a cattle strain [7, 8]. To date, reported hosts of *E. ortleppi* include cattle, camels, pigs, goats, sheep, wild mammals (including oryx, douc langur, crested porcupines and spotted deer) and humans in many regions of Europe, Africa, the Middle East, South Asia, and Latin America [17]. Shi et al. [17] reported in 2019 the first case of a human *E. ortleppi* infection in Rongshui County, Guangxi Zhuang Autonomous Region, a region of southwestern China previously considered non-endemic. This particular patient had no travel or work history related to any clearly identified epidemic areas. Here, we report a case of *E. ortleppi* infection in a similar area located in Guizhou Province, bordering the Guangxi is reported. As this constitutes a highly unusual phenomenon, we studied the pathology, phylogeny, and epidemiology of this *E. ortleppi* infection case.

## Methods

### Clinical examination and morphological observation

Herein, we reported the case of a 19-year-old female patient who resides in a village of Anshun City, Guizhou, China. She presented first in May 2019 with intermittent distention and pain in the upper abdomen for more than 10 days without evident inducements, such as fever, cough, and vomiting or diarrhoea. On 4 July, she visited the People's Hospital of Anshun City, and routine blood examination and computed tomography (CT) were performed for pathological diagnosis. A surgical treatment plan was implemented, and the cyst including wall and its entire contents of the entire lesion was removed during the implementation. The cystic lesion was harvested after extirpation and fixed it in a 10% formalin solution, subsequently produced paraffin-embedded sections that were stained with hematoxylin–eosin (HE) and observed under the microscope at 40 × magnification for pathological diagnosis.

### Molecular identification

The total DNA of the paraffin-embedded specimens was extracted using the QIAamp® DNA FFPE Tissue Kit (56,404, Qiagen, Hilden, Germany) according to the instructions of the manufacturer. We then amplified two mitochondrial DNA (mtDNA) genes; part of cytochrome c oxidase subunit 1 (*cox*1) and nicotinamide dehydrogenase subunit 1 (*nad*1). The sequences of the primers used were F/COI (5′-TTG AAT TTG CCA CGT TTG AAT GC-3′) and R/COI (5′-GAA CCT AAC GAC ATA ACA TAA TGA-3′) [18], JB11 (5′- AGA TTC GTA AGG GGC CTA ATA-3′) and JB12 (5′-ACC ACT AAC TAA TTC ACT TTC-3′) [8], respectively. We prepared a 50 μl polymerase chain reaction (PCR) reaction volume consisting of 25 μl of premix *Taq* polymerase (RR902A, Takara, Dalian, China), 20 μl of RNase-free water (9012, Takara, Dalian, China), 1.5 μl of each primer (10 pmol/μl) (Sangon Biotech Co. Ltd., Shanghai, China), and 2 μl of extracted DNA. Two PCR reactions were performed: 94 °C for 5 min, followed by 35 cycles (denaturation at 94 °C for 30 s, annealing at 52 °C for 45 s for *cox*1 and 50 °C for 30 s for *nad*1, extension at 72 °C for 1 min), and an extension step at 72 °C for 10 min, finally saved at 4 °C. The PCR products were cloned and sequenced at BGI-Write Co., Ltd. (Shanghai, China). The resulting sequences were BLAST searched in the NCBI database to identify the species.

### Phylogenetic analyses

A total of 49 sequences (27 for the *cox*1 gene, and 22 for the *nad*1 gene), including all longer length *E. ortleppi* sequences obtained in this study and those retrieved from the GenBank (other strains or species of *Echinococcus* spp.) were used for phylogenetic analysis. The sequences were edited using the Clustal X2 [19] and MAGE 7 [20] software. The substitution saturation of the sequence matrix was tested using DAMBE 5 [21]. We used jModelTest v.2.1.4 [22] to select the best-fit models for nucleotide substitution. Finally, we used the mitochondrial gene of *Taenia solium* as outgroups to construct Bayesian inference trees of the 875-bp *cox*1 and 520-bp *nad*1 genes using MrBayes 3.2.4 [23]. A haplotype network diagram was drawn using Network 5.0 (http://www.fluxus-engineering.com) to analyze the phylogenetic relationship between *E*. *ortleppi* isolated in this study and those reported worldwide.

### Epidemiological investigation

A detailed survey of the occupation, residence change, travel history, and dog contact experience of the patient, as well as the livestock trade and slaughtering situation around her residence, were conducted through field visits and communication with the patient and her families in November 2019 to determine the sources and time of infection. Additionally, we collected six samples of dog feces from around the residence of the patient to test for *Echinococcus* via a copro ELISA test using a detection kit for *Echinococcus* antigen in dog feces (Combined Biotech Co., Ltd., Shenzhen, China).

## Results

### Pathological characteristics

General medical examination including body temperature (36.9 ºC), pulse (80 /min), blood pressure (130/70 mmHg, 1 mmHg = 0.133 kPa), and respiratory rate (20 breaths/min) of the patient showed values within normal limits. Routine blood examination showed a neutrophil count (6.64 × 10^9^ / L), which was slightly higher than the normal range (1.8‒6.3 × 10^9^ /L), a lymphocyte ratio (17.6%) that was lower than the normal range (20‒50%), the eosinophil count (0.32 × 10^9^ /L) and other measurements were normal (Table 1).

**Table 1:** Results of routine blood examination of the patient in Anshun City, Guizhou Province, China

Codes	Description	Results	Reference range
WBC	White blood cell count	8.90	3.5‒9.5 × 10^9^ /L
RBC	Red blood cell count	4.71	3.8‒5.1 × 10^12^ /L
PLT	Platelet count	241.0	125‒350 × 10^9^ /L
HGB	Hemoglobin	144	115‒150 g/L
NEUT#	Neutrophil count	6.64↑	1.8‒6.3 × 10^9^ /L
NLR%	Neutrophil ratio	72.5	40‒75%
LYMPH#	Lymphocyte count	1.57	1.1‒3.2 × 10^9^ /L
LYMPH%	Lymphocyte ratio	17.6↓	20‒50%
MONO#	Monocyte count	0.52	0.1‒0.6 × 10^9^ /L
MONO%	Monocyte ratio	5.9	3‒10%
EO#	Eosinophil count	0.32	0.02‒0.52 × 10^9^ /L
EO%	Eosinophil ratio	3.6	0.4‒8%
BASO#	Basophil count	0.03	0‒0.06 × 10^9^ /L
BASO%	Basophil ratio	0.4	0‒1.0%

**Fig. 1:**
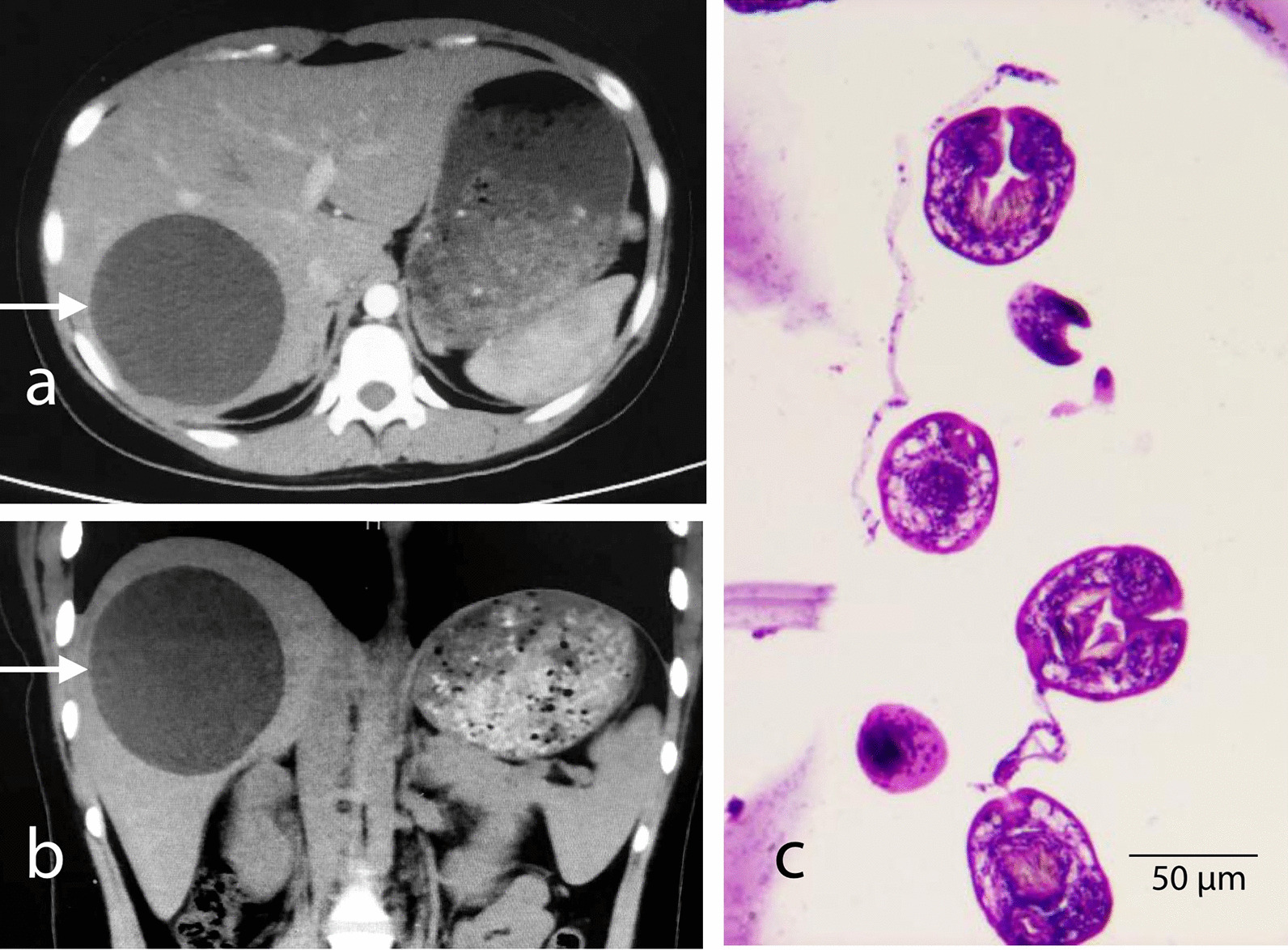
Abdominal CT **a**, **b** revealed a circular image in the right lobe of the liver later shown to be a hydatid cyst of about 8 cm in diameter. Microscopy (40 ×) of the paraffin-embedded contents after HE staining showed *Echinococcus* protoscoleces (**c**)

Abdominal CT images revealed that the right lobe of the liver had a spherical low-density shadow with a diameter of approximately 8 cm, the density was uniform, the boundary was clear and the cyst wall was not visible. According to the *Manual by the World Health Organization (WHO) and the World Organization for Animal Health (OIE) on Echinococcosis in Humans and Animals* [24], the CE classification of the finding was that the cyst was of the CL type with a medium size (5‒10 cm diameter). The HE staining revealed *Echinococcus* protoscoleces in the cyst contents that were confirmed to be fertile (Fig. 1).

### Molecular identification

We sequenced an 875-bp nucleotide sequence numbered as ‘8D21’ for the *cox*1 gene and a 520-bp sequence numbered as ‘JD11’ for the *nad*1 gene, which showed 99.66% and 99.81% identify to the two referential *E. ortleppi* sequences (accession numbers: MK165232 and MN058592, respectively) using BLAST on NCBI. Therefore, it was identified that the isolate in this study was *E. ortleppi* species*.* We submitted this information under accession numbers MZ190835 and MZ190836 (the sequences data would be released on December 21, 2021), respectively, to GenBank, the annotated collection of all publicly available DNA sequences at the National Institutes of Health (NIH) in the USA.

### Phylogenetic relationships

**Fig. 2:**
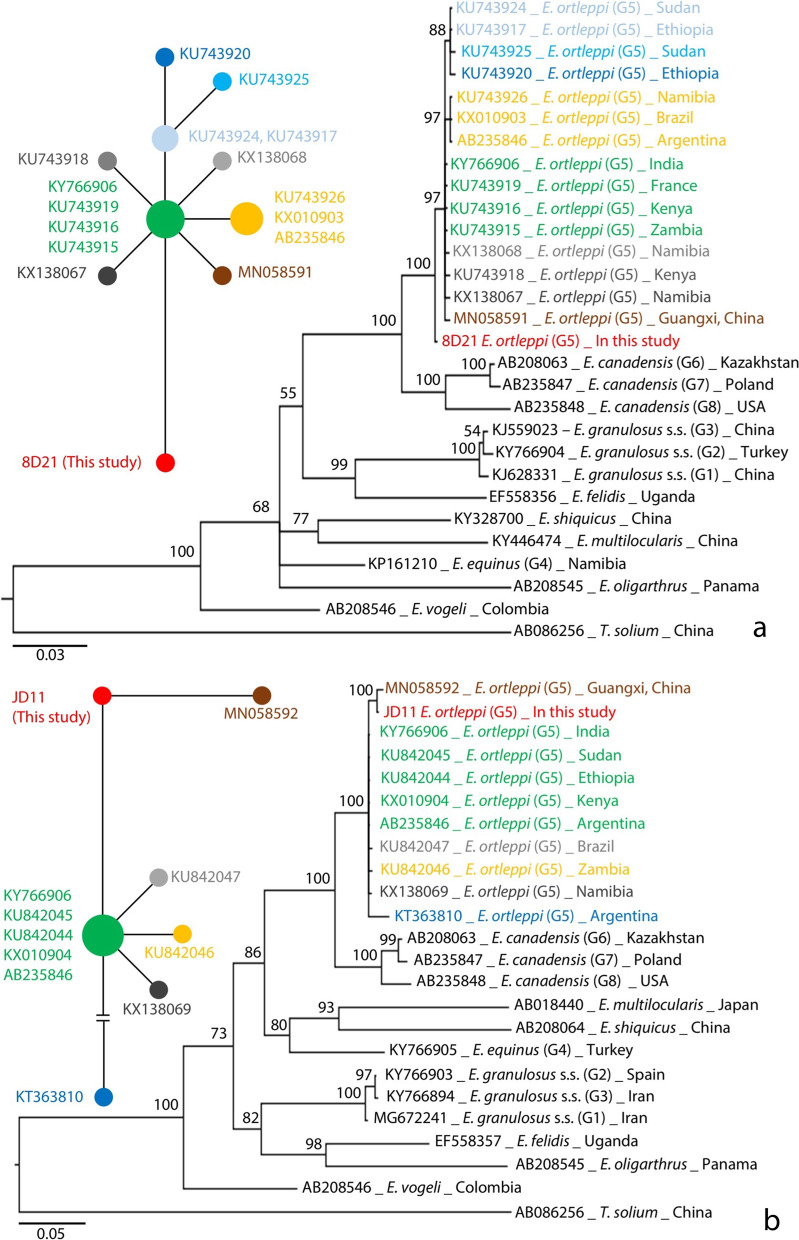
The Bayesian phylogenetic trees and haplotype networks based on mtDNA genes *cox*1 **a** and *nad*1 (**b**). The ‘GRT + G + I’ and ‘HKY + G’ substitution models with a 1000-generation sampling interval from 10 000 000 generations of Markov Chain Monte Carlo (MCMC) were set to use Bayesian inferring, respectively. The size of the circle in the network represents the number of sequences of the *E. ortleppi* haplotype, and the distance between the centers of the circles shows the variation between two haplotypes. The circle color in the network diagrams corresponds to the color of sequence names on the trees

The Bayesian phylogenetic trees of *Echinococcus* spp. based on *cox*1 and *nad*1 genes showed that the isolates in this study belonged to *E. ortleppi*, which is separated from other strains of *E. granulosus* s.l. and other species of *Echinococcus*. Parsimony network diagrams of *E. ortleppi cox*1 and *nad*1 genes revealed the variation between the haplotypes isolated in China and the other global haplotypes. Moreover, the haplotypes were shown to be different between the *E. ortleppi* reported in the two provinces of Guizhou, and Guangxi, China (Fig. 2).

### Epidemiologic history

The patient was born in July 2000 in a village of Anshun City, Guizhou Province, and lived there up to the age of nine years. After that, from September 2009, she moved to Ningbo City (Zhejiang Province) and lived until August 2012. She then moved to Pucheng County (Nanping City, Fujian Province), where she lived for three years. During this time, she made a short trip to Shanghai City in 2014. In September 2015, she traveled back to her birthplace, and lived there until the time of the survey. Based on the results of our investigation, she never visited any area identified as endemic for echinococcosis, and she had no contact with dogs from when she lived away from her birthplace.

Additionally, a cattle slaughterhouse was operating from 2005 to 2013 in the village where she was born, and the cattle were mainly brought from the local areas and Guangxi, meanwhile, dogs were always kept in her home. However, in 2019, all six samples of dog faeces collected from around the residence of the patient were negative based on an ELISA detection kit for *Echinococcus* coproantigens. Therefore, we speculate that there a possible chain of transmission of *Echinococcus* may exist locally before 2013, which consists of the essential intermediate hosts (the cattle from the slaughterhouse) and the definitive hosts (the dogs from her home). If so, the patient could have been infected with *E. ortleppi* during this period (from 2004 to 2009), particularly as the latent period of this CE disease was suggested to be 10‒14 years.

A more detail spatiotemporal overview of the patient's life history is shown in an additional figure (see Additional file 1).

## Discussion

The patient presented with only abdominal pain without any other symptoms. The most important indicators of the parasitic infection, i.e. count and percentage of eosinophil granulocytes were normal; the cysts had no typical CE (CE1‒CE5) features seen in the CT imagery [24], such as an evident cyst wall structure consistent with the results of a previous study [6, 17]. These preoperative clinical features did not reveal whether the liver cyst was caused by *Echinococcus* since it was very similar to a congenital liver cyst. Indeed, Maravilla et al. [27] similarly reported a misdiagnosed case of *E. ortleppi* infection in Mexico. Therefore, differential diagnoses require further diagnostic approaches. The formulation of the surgical plan was affected since protoscoleces could result in severe secondary infections if the cyst turned out to be parasitic in origin and run the risk of rupture during the operation. In the present study, the cyst could be removed without breaking and the subsequent microscopy showed protoscoleces proving it was a fertile *Echinococcus* cyst.

Molecular diagnosis results revealed that *E. ortleppi* caused the infection. We speculate that the type CL cyst presentation was either abnormal or that *E. ortleppi* infection tended to manifest in humans with type CL characteristics. This requires a large sample size to elucidate, which is available in this case. This is the second report on CE caused by *E. ortleppi* infection in China. In accordance with the first case, it occurred in an area considered to be free of echinococcosis [17]. It has been documented that the main epidemic areas of echinococcosis in the pastoral and semi-pastoral regions of the western China show a large number of *Echinococcus* genotype G1, with sporadic G3 and G6 type presence but without *E. ortleppi* (G5) being reported [12–14]. The mitochondrial gene sequences of *E. ortleppi* obtained from the two cases in China are new haplotypes differing from those reported in other regions in the world. This led to the suspicion that the cases were due to local infections, and that there may be, or has been, a chain of transmission of *E. ortleppi* in the areas of south-western China. This calls for an investigation into the epidemiology and molecular phylogeny of echinococcosis in the southwestern China regions including Guangxi, and Guizhou provinces to search for a potential life cycle of *E. ortleppi*.

The distribution of *E. multilocularis* is obviously regional. It is commonly found in high latitude regions of the northern hemisphere (such as Canada, Central Europe, Russian Federation, Mongolia, Hokkaido of Japan and Northern China), alpine regions (such as the Qinghai-Tibet Plateau of China) and permafrost regions (such as Alaska of USA) [28]. However, there is an obvious difference between *E. ortleppi* and *E. multilocularis,*according to the statistical results of Shi et al. [17], *E. ortleppi* infection cases in humans or other intermediate hosts have been reported in Asia, Africa, America and Europe, but, the countries with more than 10 cases reported are mainly in the low-latitude tropical area, such as Brazil (536 cases) in South America [29–33], Ethiopia (13 cases) [29, 34], Kenya (91 cases) [29, 35, 36, Sudan (17 cases) [29, 37], Zambia (53 cases) [29] and Namibia (38 cases) [29] in Africa, and India (12 cases) [2, 38, 39] in South Asia. So, it was inferred that there may be a stable tropical strain of *Echinococcus* in the long-term environmental adaptive evolution. For China, given the lack of genetic data, it remains elusive whether the *E. ortleppi* in China originated from the cattle trade across national borders (tropics) in the ancient era or emerged through natural a local selection processes operating over a long time.

Considering that both Guizhou and Guangxi are southern province or region of China, which are close to tropical areas in geographical locations. The warm climate conditions may be favorable for *E. ortleppi* survival and reproduction, so, here may be a risk of echinococcosis infection. Therefore, we strongly believe that more attention should be paid to echinococcosis in the previously considered non-endemic areas in China and the improve dissemination of health education to the people in the potentially endemic areas. Moreover, the quarantine and management of animals should be enhanced, especially in livestock slaughterhouses, to prevent emerging infections.

However, this investigation only focused on one case, and discussed the transmission chain of *E. ortleppi* in southwest China in combination with another case previously reported [17]. The sample size was very limited, which also led to the limitations of the inference in this study. According to a report by Han et al. [40], at of the end of 2017, a total of 15 cases and 6 cases were reported in Guizhou Province and Guangxi Zhuang Autonomous Region, respectively. Detailed reports about the infection sources and routes of these cases are still scarce, which restricts the understanding of distribution and transmission of *E. ortleppi* in China.

## Conclusions

In this study, a fertile cyst was formed on the patient’s liver, and it was confirmed as the CE with CL type caused by *E. ortleppi*. Differentiated inheritance variation of parasite and blank history to stay in the epidemic area identified of patient showed that it might be a locally infected case. To date, the only two *E. ortleppi* infections found in southwestern China represent a potential transmission pattern, which requires detailed field investigations and laboratory experiments to understand. Moreover, chronic parasitic infectious diseases of this kind pose a high health risk in humans since its atypical pathological features might result in misdiagnosis and spill into other organs during surgery. The infection features, life cycle, and transmission mode of *E. ortleppi* may be different from those of other *Echinococcus* spp.; therefore, more studies on the pathology, molecular biology, and epidemiology should be conducted.

## Supplementary Information

**Additional file 1.** The traced location and time of the patient. **a** The roadmap for relocation and travel. **b** Life history timeline. Statistics of endemic areas originate from the Technical Scheme for Control of Echinococcosis (Edition 2019) [25] and a nationwide sampling survey [26]. Rongshui County is the place of the first description of *E. ortleppi* infection in China. Anshun City is the birthplace of the patient, and the settlement she lived before September 2009 and after September 2015. Ningbo City is the second settlement (from September 2009 to August 2012), Pucheng County is the third settlement (from August 2012 to September 2015), and Shanghai City is the destination of a short tour in 2014.

## Data Availability

The data used in this study are available, if necessary, please contact first author (XW) or corresponding author (WPW).

## Abbreviations

CE: Cystic echinococcosis
s.l.: Sensu lato
s.s.: Sensu stricto
CT: Computed Tomography
*cox*1: Cytochrome c oxidase subunit 1
*nad*1: Nicotinamide dehydrogenase subunit 1
