# Supplementary figures and images for: The pathology, phylogeny, and epidemiology of Echinococcus ortleppi (G5 genotype): a new case report of echinococcosis in China
Source: Infect Dis Poverty. 2021 Nov 6;10:130. doi: 10.1186/s40249-021-00907-3 (PMC8572459; doi:10.1186/s40249-021-00907-3)

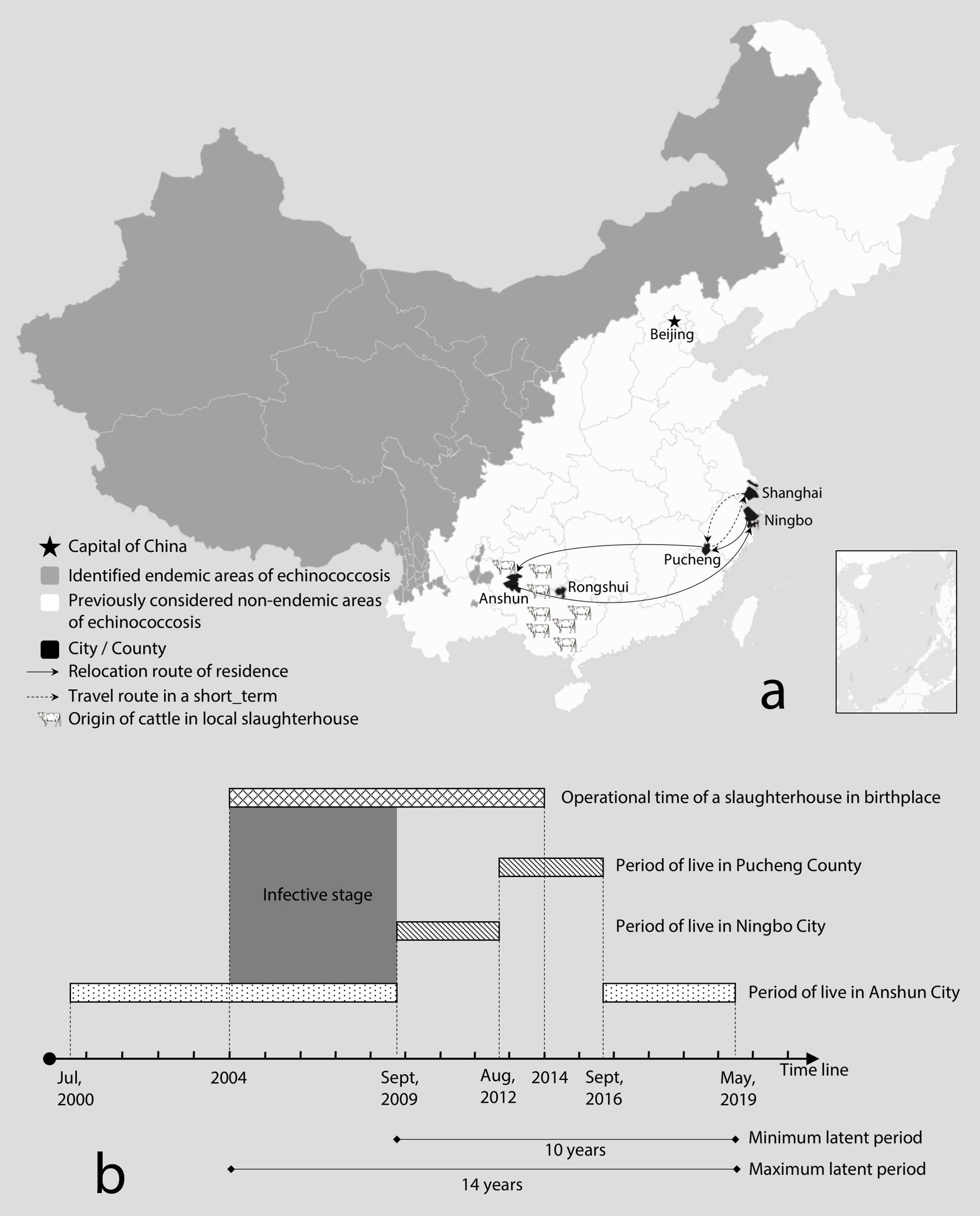

Supplement: Supplementary file 1 — Additional file 1. The traced location and time of the patient. a The roadmap for relocation and travel. b Life history timeline. Statistics of endemic areas originate from the Technical Scheme for Control of Echinococcosis (Edition 2019) [25] and a nationwide sampling survey [26]. Rongshui County is the place of the first description of E. ortleppi infection in China. Anshun City is the birthplace of the patient, and the settlement she lived before September 2009 and after September 2015. Ningbo City is the second settlement (from September 2009 to August 2012), Pucheng County is the third settlement (from August 2012 to September 2015), and Shanghai City is the destination of a short tour in 2014. [file 40249_2021_907_MOESM1_ESM.jpg]
